# DOES DENTAL TRAUMA IN EARLY CHILDHOOD HAVE THE POTENTIAL TO AFFECT THE QUALITY OF LIFE OF CHILDREN AND FAMILIES?

**DOI:** 10.1590/1984-0462/2021/39/2019329

**Published:** 2020-11-06

**Authors:** Diego Patrik Alves Carneiro, Patricia Rafaela dos Santos, Heloísa Cristina Valdrighi, Marcelo de Castro Meneghim, Silvia Amélia Scudeler Vedovello

**Affiliations:** a Hermínio Ometto Foundation, Araras, SP, Brazil.; b Universidade Estadual de Campinas, Piracicaba, SP, Brazil.

**Keywords:** Quality of life, Tooth injuries, Dental caries, Malocclusion, Qualidade de vida, Traumatismos dentários, Cárie dentária, Má oclusão

## Abstract

**Objective:**

To evaluate the influence of dental trauma on oral health-related quality of life (OHRQoL) of children and their families.

**Methods:**

A total of 571 children aged five years were randomly selected at public schools. Trauma was clinically evaluated in accordance with the Andreasen classification. Caries experience in the anterior region and increased overjet were determined according to the World Health Organization criteria. The Early Childhood Oral Health Impact Scale (ECOHIS) was answered by the parents and used to evaluate OHRQoL. In addition, this questionnaire has aspects related to socioeconomic status. Simple logistic regression was performed, and the raw *Odds Ratios* with the respective 95% confidence intervals (95%CI) were estimated. The variables with p<0.20 were tested in multiple logistic regression models, and those with p≤0.05 remained in the model and the adjusted odds ratio with respective 95%CI was estimated.

**Results:**

Income showed a magnitude of association of 1.56 and 2.70 with the OHRQoL of children and families, respectively. The avulsion variable showed 9.65- and 8.25-times greater chance of influencing the OHRQoL of children and families, respectively. The experience of caries showed 3.80- and 2.42-times greater chance of influencing the OHRQoL of children and families, respectively.

**Conclusions:**

Dental trauma did not influence OHRQoL of children and their families negatively. However, avulsion and caries experience in low-income families was associated with a negative perception of OHRQoL.

## RESUMO

**Objetivo:**

Avaliar a influência do traumatismo dentário na qualidade de vida relacionada à saúde bucal (QVRSB) de crianças e suas famílias.

**Métodos:**

571 crianças de 5 anos de idade foram aleatoriamente selecionadas de escolas públicas. O trauma foi avaliado clinicamente de acordo com a classificação de Andreasen. A experiência de cárie na região anterior e a presença de *overjet* foram determinadas com base nos critérios da Organização Mundial da Saúde. A versão brasileira do questionário ECOHIS (Escala de Impacto na Saúde Oral na Primeira Infância) avaliou a QVRSB e foi respondida pelos pais; além disso, foram avaliados aspectos socioeconômicos. Realizou-se uma regressão logística simples, bem como as razões de chances brutas com os respectivos intervalos de confiança de 95%. As variáveis ​​com p <0,20 foram testadas nos modelos de regressão logística múltipla, e aquelas com p≤0,05 permaneceram no modelo.

**Resultados:**

A renda mostrou uma magnitude de associação de 1,56 e 2,70 com a QVRSB das crianças e famílias, respectivamente. A variável avulsão apresentou chance 9,65 e 8,25 vezes maior de influenciar a QVRSB de crianças e famílias, respectivamente. A experiência de cárie mostrou chance 3,80 e 2,42 vezes maior de influenciar a QVRSB de crianças e famílias, respectivamente.

**Conclusões:**

O trauma dental não influenciou negativamente a QVRSB das crianças e suas famílias. Entretanto, especificamente a avulsão, e a experiência de cárie nos dentes anteriores em famílias de baixa renda estiveram associadas a uma percepção negativa da QVRSB.

## INTRODUCTION

Oral health-related quality of life indicators (OHRQoL) must be combined with clinical evaluation to establish priorities in Oral Health.^1^ Traumatic dental lesions are known to have an early negative impact on OHRQoL, right away in early childhood.^2^ ^,^ ^3^ ^,^ ^4^ New and repeated trauma may occur throughout the child’s growth and development, affecting dental, periodontal, bone, and soft tissue structures.^5^ ^,^ ^6^

Dental trauma may bring negative consequences to the child’s life, such as pain and difficulty in chewing, as well as affect dentofacial esthetics and, therefore, the individual’s social interaction, depending on the severity of the sequelae.^5^ ^,^ ^7^ ^,^ ^8^ ^,^ ^9^ Enamel fracture is the most common dental trauma in early childhood, but this normally presents minimal consequences, and will rarely be the reason for esthetic complaints. Thus, this type of trauma may go unnoticed by the child and parents, and/or caregivers.^10^ In addition, trauma has been studied to a larger extent in mixed dentition, but not in the deciduous dentition and under the perspective of the child’s family.

Therefore, dental trauma and clinical conditions such as dental caries and severe malocclusion are those perceived by the parents when they become esthetically evident or when they are associated with pain.^5^ ^,^ ^11^ ^,^ ^12^ For directing oral health protocols related to trauma, it is fundamental to evaluate the parents’ reports.^5^ ^,^ ^8^ ^,^ ^12^ In this context, the aim of this study was to evaluate the influence of dental trauma on oral health-related quality of life (OHRQoL) of children and their families, adjusted by clinical and socioeconomic conditions.

## METHOD

This is a cross-sectional epidemiological study conducted with 5-year old children, enrolled at the 17 municipal schools in Araras (São Paulo, Brazil). A minimum sample of 549 individuals was calculated; considering a level of significance of 5%, test power of 80%, *Odds Ratio* of 1.6, and response rate of 50% in the unexposed group. The sample was stratified according to administrative district and, in the first phase, schools were randomly selected. In the second phase, children were selected for the sample using a simple randomization procedure. Classrooms were randomly selected at the schools, and children were randomly selected from the classes. Only children whose parents authorized the examination were included in the study; those with absence of previous orthodontic treatment or not undergoing this treatment at the time of the study, and those who were free of systemic or neuromotor diseases or had difficulties with communication. Children who presented absence of anterior teeth due to natural exfoliation, loss for other reasons or presence of enamel defects were not included in this study, as this was considered as a confounding factor. At the time of the examination, 1,004 children in the target age group were enrolled in municipal schools. Considering the sample size and selection criteria, 571 schoolchildren and their families participated in the study, which had previously been approved by the Research Ethics Committee (Report Number 1.735.990).

Parents were invited to answer the Early Childhood Oral Health Impact Scale (ECOHIS) questionnaire validated for the Brazilian population.^13^ The questionnaire, composed of 13 questions, included nine addressing the impact on children, and four addressing impact on the family. Respondents used a rating scale from 0 to 5, where: 0=never, 1=hardly ever, 2=occasionally, 3=often, 4=very often, and 5=do not know. Total scores ranged from zero to 52. The zero score indicated no impact on quality of life. Scores higher than zero indicated negative impact on quality of life.

In addition, parents were asked about aspects such as their educational level (up to 8th grade completed, or >8th grade completed) and family income (≤US$ 620 or >US$ 620).

Clinical exams were performed at the school, in a room under natural light, with the help of wooden spatulas and gauze, by a single, trained and calibrated examiner. Inter-examiner Kappa coefficients were calculated after calibration between the gold standard professional and the dentist. The values were higher than 0.89, 0.81 and 0.92 for traumatic dental injury (TDI), caries and malocclusion, respectively.

Dental caries experience was evaluated in accordance with the criteria recommended by the World Health Organization.^14^ For data analysis, only the maxillary and mandibular anterior teeth - canine to canine - were considered and dichotomized into presence or absence of disease.

To evaluate overjet, the Foster and Hamilton’s^15^ index was used. The overjet was evaluated by the horizontal distance between the upper and lower incisors. No distance between maxillary and mandibular incisors was defined as normal overjet (0 mm). Increased overjet was recorded when the distance was >2 mm, and anterior crossbite was recorded when the distance was <0 mm. For the analysis, the overjet data were dichotomized into presence or absence thereof.^15^

TDI were evaluated in the maxillary and mandibular deciduous teeth - from canine to canine - and classified by the criteria proposed by Andreasen et al.: enamel fracture, enamel-dentin fracture with and without pulp exposure, and avulsion. The presence of tooth color change was also evaluated.^16^

After calculating the prevalence of children with dental trauma and the 95%CI, a frequency distribution table of the exploratory variables was built in relation to the three outcomes (Child Impact Section, Family Impact Section and General Score Impact Section). Then simple logistic regression models were adjusted, estimating gross *Odds Ratios* and 95%CI. The chi-square test was used for bivariate analysis with 95%CI. To analyze the possible confounding effect, the variables with p<0.20 in simple analyses were selected to compose the hierarchical multiple logistic regression model. The model was built considering two levels: level 1 (demographic and socioeconomic factors) and level 2 (clinical aspects). Initially, the variables of the first level were adjusted to others of the same level, remaining in the model when p≤0.05. The second level variables were adjusted to others of the same level, remaining in the final model the variables when p≤0.05 after the adjustments. From the final model, the adjusted *Odds Ratios* and 95%CI were estimated. The analyses were performed in the program R (R Core Team, Vienna, Austria).

## RESULTS

Table 1 presents descriptive data with relative and absolute frequencies of independent variables on OHRQoL. A total of 571 children were examined. Of these, 48.7% were girls, 63.2% of families had a monthly family income ≤US$ 620, and, 25.7% of mothers and 19.1% of fathers had more than 10 years of study. The prevalence of dental trauma was 34.5%. Of these, 30.5% of the children had crown fracture and 90.5% had no change in color. Caries experience in the anterior region was present in 8.1% of children evaluated, and increased overjet was seen in 31,5% of them.

**Table 1:** Frequency and percentage of participants in each category for outcomes Child Impact Section, Family Impact Section and General Score Impact Section.

Level	Variable	Category	N (%^$^)	Child Impact Section		Family Impact Section		General Score Impact Section	
				Without impact	With impact^#^	Without impact	With impact^#^	Without impact	With impact^#^
				n (%)	n (%)	n (%)	n (%)	n (%)	n (%)
Demographic and socioeconomic factors	Sex	Male	293 (51.3%)	184 (62.8%)	109 (37.2%)	251 (85.7%)	42 (14.3%)	170 (58.0%)	123 (42.0%)
		Female	278 (48.7%)	177 (63.7%)	101 (36.3%)	238 (85.6%)	40 (14.4%)	167 (60.1%)	111 (39.9%)
	Race	White	398 (69.7%)	258 (64.8%)	140 (35.2%)	345 (86.7%)	53 (13.3%)	243 (61.1%)	155 (38.9%)
		Non-white	173 (30.3%)	103 (59.5%)	70 (40.5%)	144 (83.2%)	29 (16.8%)	94 (54.3%)	79 (45.7%)
	Income	≤US$ 620	361 (63.2%)	213 (59.0%)	148 (41.0%)	295 (81.7%)	66 (18.3%)	196 (54.3%)	165 (45.7%)
		>US$ 620	210 (36.8%)	148 (70.5%)	62 (29.5%)	194 (92.4%)	16 (7.6%)	141 (67.1%)	69 (32.9%)
	Schooling - Father	Up to10 years	462 (80.9%)	292 (63.2%)	170 (36.8%)	397 (85.9%)	65 (14.1%)	273 (59.1%)	189 (40.9%)
		Over 10 years	109 (19.1%)	69 (63.3%)	40 (36.7%)	92 (84.4%)	17 (15.6%)	64 (58.7%)	45 (41.3%)
	Schooling - Mother	Up to10 years	424 (74.3%)	265 (62.5%)	159 (37.5%)	361 (85.1%)	63 (14.9%)	247 (58.3%)	177 (41.7%)
		Over 10 years	147 (25.7%)	96 (65.3%)	51 (34.7%)	128 (87.1%)	19 (12.9%)	90 (61.2%)	57 (38.8%)
Clinical Aspects	Presence of trauma	Absent	374 (65.5%)	235 (62.8%)	139 (37.2%)	322 (86.1%)	52 (13.9%)	219 (58.6%)	155 (41.4%)
		Present	197 (34.5%)	126 (64.0%)	71 (36.0%)	167 (84.8%)	30 (15.2%)	118 (59.9%)	79 (40.1%)
	Type of trauma	Absent	374 (65.5%)	235 (62.8%)	139 (37.2%)	322 (86.1%)	52 (13.9%)	219 (58.6%)	155 (41.4%)
		Enamel fracture	174 (30.5%)	116 (66.7%)	58 (33.3%)	150 (86.2%)	24 (13.8%)	108 (62.1%)	66 (37.9%)
		Enamel-dentin fracture	16 (2.8%)	9 (56.3%)	7 (43.8%)	14 (87.5%)	2 (12.5%)	9 (56.3%)	7 (43.8%)
		Avulsion	7 (1.2%)	1 (14.3%)	6 (85.7%)	3 (42.9%)	4 (57.1%)	1 (14.3%)	6 (85.7%)
	Color alteration	No	517 (90.5%)	332 (64.2%)	185 (35.8%)	445 (86.1%)	72 (13.9%)	311 (60.2%)	206 (39.8%)
		Yes	54 (9.5%)	29 (53.7%)	25 (46.3%)	44 (81.5%)	10 (18.5%)	26 (48.1%)	28 (51.9%)
	Experience of caries	Absent	525 (91.9%)	346 (65.9%)	179 (34.1%)	456 (86.9%)	69 (13.1%)	323 (61.5%)	202 (38.5%)
		Present	46 (8.1%)	15 (32.6%)	31 (67.4%)	33 (71.7%)	13 (28.3%)	14 (30.4%)	32 (69.6%)
	Overjet increased	Absent	391 (68.5%)	245 (62.7%)	146 (37.3%)	337 (86.2%)	54 (13.8%)	230 (58.8%)	161 (41.2%)
		Present	180 (31.5%)	116 (64.4%)	64 (35.6%)	152 (84.4%)	28 (15.6%)	107 (59.4%)	73 (40.6%)

^$^Column percent; ^#^reference category for the outcome variable.

The association between independent variables and the parents’ perception of the impact of oral health on the child’s, family’s and total quality of life assessed by the ECOHIS is shown in Table 2. Regarding demographic and socioeconomic variables, family income showed a magnitude of association of 1.56 (95%CI 1.07-2.27), 2.70 (95%CI 1.50-4.86) and 1.64 (95%CI 1.14-2.36) with children’s quality of life, family, and overall ECOHIS score, respectively. Regarding clinical aspects, avulsion showed a 9.65 (95%CI 1.14-82.10) and 8.25 (95%CI 1.75-38.81) times greater chance of influencing the quality of life of children and families, respectively. The experience of caries showed a 3.80 (95%CI 1.99-7.28), 2.42 (95%CI 1.20-4.89) and 3.47 (95%CI 1.79-6.70) times greater chance of influencing the children’s quality of life, family, and overall ECOHIS score, respectively.

**Table 2:** Analysis of the associations between the independent variables and the parents’ perception of the impact of oral health on the child’s, family and total quality of life, assessed by the ECOHIS instrument.

Level	Variable	Category	Child Impact Section		Family Impact Section		General Score Impact Section	
			OR^$^ crude (CI^#^)	OR^$^ adjusted (CI^#^)	OR^$^ crude (CI^#^)	OR^$^ adjusted (CI^#^)	OR^$^ crude (CI^#^)	OR^$^ adjusted (CI^#^)
Demographic and socioeconomic factors	Sex	Male	Ref		Ref		Ref	
		Female	0.96 (0.68-1.35)		1.00 (0.63-1.60)		0.92 (0.66-1.28)	
	Race	White	Ref		Ref		Ref	
		Non-white	1.25 (0.87-1.81)		1.31 (0.80-2.15)		1.32 (0.92-1.89)	
	Income	≤US$ 620	**1.66 (1.15-2.38)	*1.56 (1.07-2.27)	**2.71 (1.53-4.82)	**2.70 (1.50-4.86)	**1.72 (1.21-2.45)	**1.64 (1.14-2.36)
		>US$ 620	Ref	Ref	Ref	Ref	Ref	Ref
	Schooling - Father	Up to 10 years	1.00 (0.65-1.55)		0.88 (0.50-1.58)		0.98 (0.64-1.50)	
		Over 10 years	Ref		Ref		Ref	
	Schooling - Mother	Up to 10 years	1.13 (0.76-1.67)		1.18 (0.68-2.04)		1.13 (0.77-1.66)	
		Over 10 years	Ref		Ref		Ref	
Clinical aspects	Presence of trauma	Absent	Ref		Ref		Ref	
		Present	0.95 (0.67-1.36)		1.11 (0.68-1.81)		0.95 (0.67-1.34)	
	Type of trauma	Absent	Ref	Ref	Ref	Ref	Ref	Ref
		Enamel fracture	0.84 (0.56-1.23)	0.89 (0.60-1.31)	0.99 (0.59-1.67)	1.11 (0.65-1.90)	0.86 (0.60-1.25)	0.92 (0.63-1.34)
		Enamel-dentin fracture	1.32 (0.48-3.61)	1.18 (0.42-3.32)	0.88 (0.20-4.00)	0.74 (0.16-3.42)	1.10 (0.40-3.01)	0.97 (0.34-2.74)
		Avulsion	*10.14 (1.21-85.12)	*9.65 (1.14-82.10)	**8.26 (1.80-37.95)	**8.25 (1.75-38.81)	*8.48 (1.01-71.08)	^1^7.99 (0.94-67.84)
	Color alteration	No	Ref		Ref		Ref	
		Yes	1.55 (0.88-2.72)		1.40 (0.68-2.92)			
	Experience of caries	Absent	Ref	Ref	Ref	Ref	Ref	Ref
		Present	**3.99 (2.10-7.59)	**3.80 (1.99-7.28)	**2.60 (1.31-5.19)	*2.42 (1.20-4.89)	**3.66 (1.90-7.02)	**3.47 (1.79-6.70)
	Overjet increased	Absent	Ref		Ref		Ref	
		Present	0.93 (0.64-1.34)		1,15 (0.70-1.89)		0.98 (0.68-1.40)	

^$^ *Odds Ratio*; ^#^95% confidence interval; *p<0.05; **p<0.01; ^1^p<0.06.

## DISCUSSION

In view of the relevant influence that dental trauma may cause on the quality of life of the families, especially of children in early childhood, this may be considered a public health question. However, divergences are still found in the literature.^7^ ^,^ ^10^ ^,^ ^17^ In the present study, there was no association between dental trauma and OHRQoL. The lack of association may be due to the highest prevalence of dental trauma involving only enamel fracture (30.5%), which is difficult to be perceived. When assessing the types of dental trauma separately, only avulsion was associated with a reduced ORHQoL, corroborating previous studies.^7^ ^,^ ^17^ ^,^ ^18^

The perception of parents/caregivers is directly linked to the clinical conditions that present symptoms in their children, this feature justifies the association of dental caries in the anterior region and its influence on quality of life, considering that in a situation where there is a report pain or complaint in chewing and aesthetic interference the family is affected.^7^ ^,^ ^19^ Dental caries restricted to the posterior region in early stages, since they do not cause pain, may not be noticed by the child and parents/caregivers, which reduces the influence of this condition on the quality of life. In the opposite direction, a previous study^12^ pointed out only the presence of caries in the posterior teeth as associated with negative impact on the quality of life, that is, dental caries in the posterior region were more severe.

Increased overjet was related to the presence of trauma. Although this occlusal condition presented high prevalence (31,5%) in the children evaluated, it did not negatively influence the quality of life, corroborating the findings of previous studies conducted in deciduous dentition.^8^ ^,^ ^12^ ^,^ ^20^ A hypothesis is how overjet is evaluated in the deciduous dentition stage. According to the method described by Foster and Hamilton^15^ and recommended by the WHO for evaluation at five years of age, horizontal overjet was dichotomized into normal or increased, without taking into account the severity of the clinical condition.

Low income was a factor that contributed to the negative impact on oral health-related quality of life.^9^ ^,^ ^21^ ^,^ ^22^ ^,^ ^23^ The vulnerability caused by income may be related to a lower level of access to information, and consequently little demand for the services.^22^ ^,^ ^23^ The high prevalence of dental trauma shows the need for educational and preventive programs based on the understanding of multifactorial variables related to etiology and the motivation to seek treatment.

The present study presents the limitations inherent to the cross-sectional design and the response rate of the questionnaires by parents/caregivers; however, the minimum sample size was reached.

Understanding the implications of dental trauma in an individual’s life is important for educational strategies to be established. In the pre-school stage, implications go beyond the occurrence of trauma or its clinical consequences. Therefore, the implementation of educational strategies aimed at parents/caregivers is necessary, with a strict guidance that, if dental trauma occurs, they are informed thar seeking professional management is important. Finally, a longitudinal study may show evidence of the cause-effect relationship between the identified associations. In conclusion, avulsion and caries experience in their anterior teeth caused a negative impact on the oral health-related quality of life of both the children and their families, and this was associated with low family income.
